# Plasma Free Hemoglobin and Microcirculatory Response to Fresh or Old Blood Transfusions in Sepsis

**DOI:** 10.1371/journal.pone.0122655

**Published:** 2015-05-01

**Authors:** Elisa Damiani, Erica Adrario, Michele Maria Luchetti, Claudia Scorcella, Andrea Carsetti, Nicoletta Mininno, Silvia Pierantozzi, Tiziana Principi, Daniele Strovegli, Rosella Bencivenga, Armando Gabrielli, Rocco Romano, Paolo Pelaia, Can Ince, Abele Donati

**Affiliations:** 1 Department of Biomedical Sciences and Public Health, Università Politecnica delle Marche, Torrette di Ancona, Italy; 2 Anesthesia and Intensive Care Unit, Azienda Ospedaliera Universitaria “Ospedali Riuniti”, Torrette di Ancona, Italy; 3 Department of Clinical and Molecular Sciences, Clinica Medica, Università Politecnica delle Marche, Torrette di Ancona, Italy; 4 Immunohematology and Transfusional Medicine, AOU Ospedali Riuniti, Torrette di Ancona, Italy; 5 Department of Translational Physiology, Academic Medical Center, Amsterdam, The Netherlands; Erasmus Medical Centre, NETHERLANDS

## Abstract

**Background:**

Free hemoglobin (fHb) may induce vasoconstriction by scavenging nitric oxide. It may increase in older blood units due to storage lesions. This study evaluated whether old red blood cell transfusion increases plasma fHb in sepsis and how the microvascular response may be affected.

**Methods:**

This is a secondary analysis of a randomized study. Twenty adult septic patients received either fresh or old (<10 or >15 days storage, respectively) RBC transfusions. fHb was measured in RBC units and in the plasma before and 1 hour after transfusion. Simultaneously, the sublingual microcirculation was assessed with sidestream-dark field imaging. The perfused boundary region was calculated as an index of glycocalyx damage. Tissue oxygen saturation (StO_2_) and Hb index (THI) were measured with near-infrared spectroscopy and a vascular occlusion test was performed.

**Results:**

Similar fHb levels were found in the supernatant of fresh and old RBC units. Despite this, plasma fHb increased in the old RBC group after transfusion (from 0.125 [0.098–0.219] mg/mL to 0.238 [0.163–0.369] mg/mL, p = 0.006). The sublingual microcirculation was unaltered in both groups, while THI increased. The change in plasma fHb was inversely correlated with the changes in total vessel density (r = -0.57 [95% confidence interval -0.82, -0.16], p = 0.008), De Backer score (r = -0.63 [95% confidence interval -0.84, -0.25], p = 0.003) and THI (r = -0.72 [95% confidence interval -0.88, -0.39], p = 0.0003).

**Conclusions:**

Old RBC transfusion was associated with an increase in plasma fHb in septic patients. Increasing plasma fHb levels were associated with decreased microvascular density.

**Trial Registration:**

ClinicalTrials.gov NCT01584999

## Introduction

Anaemia is common in the Intensive Care Units (ICUs) [1]. Approximately 40% of patients receive packed red blood cell (RBC) transfusions during their ICU stay [2]. The goal of blood transfusion is to increase blood oxygen (O_2_)-carrying capacity, thus restoring tissue oxygenation [3]. Although potentially life-saving in individual patients, transfusion practice was associated with increased morbidity and/or mortality in different patient populations [4, 5].

Stored packed RBCs may develop alterations over time, collectively referred to as “storage lesions”, which compromise their hemorrheological properties and O_2_-delivery capacity [6]. These include depletion of adenosine triphosphate and 2,3-diphosphoglycerate, membrane phospholipid peroxidation and vesiculation, protein oxidation, loss of deformability and increased osmotic fragility [7]. Increasing hemolysis and release of cell-free hemoglobin (fHb) were documented as a function of time during prolonged storage [8]. fHb is a potent scavenger of nitric oxide (NO), the most important endogenous vasodilator [9], and may therefore be responsible for microvascular perfusion disturbances [10].

Endothelial dysfunction and impaired microcirculatory blood flow are leading aspects in the pathophysiology of sepsis [11, 12]. Persistent microvascular alterations are associated with organ failure and death in patients with septic shock [13]. Severe deregulation in the NO system is a major cause of sepsis-induced microvascular perfusion failure [11]. Interestingly, increased plasma fHb levels are associated with higher mortality in patients with sepsis [14, 15]. A reduction in NO availability induced by the transfusion of stored RBCs may synergize with the underlying endothelial dysfunction and be responsible for tissue hypoperfusion. In the present study, we aimed to evaluate whether the transfusion of old RBCs increases plasma fHb in septic patients and how this may affect the microvascular response to blood transfusion.

## Materials and Methods

This study is a secondary analysis of a prospective randomized pilot trial whose primary aim was to evaluate the effects of fresh (<10 days storage) non-leukodepleted, fresh leukodepleted or old (>15 days storage) non-leukodepleted RBCs transfusion on the microcirculation in septic patients. A comparison between the first two groups (fresh non-leukodepleted and fresh leukodepleted) was focused on the potential role of leukocyte reduction and reported previously [16]. Herein, we focus our attention on the role of storage and report the comparison between fresh non-leukodepleted and old non-leukodepleted groups. Data related to the fresh RBC group in this report have been already presented in [16] as “fresh non-leukodepleted” group.

The study protocol was approved by the local Ethics Committee of “Azienda Ospedaliera Universitaria (AOU) Ospedali Riuniti” of Ancona in Italy (NCT01584999, www.clinicaltrials.gov). Written informed consent was obtained from the enrolled patients or their next of kin.

### Patients

Between February 2011 and 2012, adult patients admitted to the 12-bed Intensive Care Unit of the AOU Ospedali Riuniti of Ancona with sepsis, severe sepsis, or septic shock as diagnosed according to standard criteria [17] and requiring blood transfusion for Hb levels <8 g/dL or as indicated by the attending physician (in accordance with the local hospital guidelines) were eligible to participate. Exclusion criteria were: age <18 years, previous blood transfusions during their ICU stay, previous history of coagulation disorders, cardiogenic or hemorrhagic shock, pregnancy, factors impeding the sublingual microcirculation evaluation (oral surgery, maxillofacial trauma). Sedation and analgesia were provided according to individual needs, as well as the type of fluids infused (crystalloids and colloids) and adrenergic agents (norepinephrine, dobutamine). The goal was to maintain a mean arterial pressure of 65 mmHg as recommended by the international guidelines of the Surviving Sepsis Campaign (2008) [18]. Fluid infusion and furosemide treatment were titrated according to individual needs, in order to maintain an adequate urine output (>0.5 mL/kg/h) [18].

### Interventions

The enrolled patients from the primary study randomly received either fresh non-leukodepleted RBCs (<10 day storage), fresh leukodepleted RBCs (<10 day storage) or old non-leukodepleted RBCs (>15 days storage). Blood product randomisation was performed through sealed envelopes by a physician at the blood bank, who blindly provided the blood bags to the ICU; neither the attending physician nor the investigators nor the patients were aware of the type of RBCs transfused. Herein, we present data from the fresh non-leukodepleted and old non-leukodepleted groups, hereafter referred to as fresh RBC and old RBC groups.

### Basic haemodynamic and blood gas parameters

All measurements were performed in each patient immediately before and 1 hour after the end of all RBC transfusions; these time points were chosen on the basis of those reported in previous studies [19–21]. We recorded temperature (T), heart rate (HR) and mean arterial pressure (MAP). Arterial blood samples were withdrawn in order to assess Hb level, whole blood cell counts, blood gases (pH, paO₂, paCO₂, SaO₂, paO₂/FiO₂, HCO₃^-^, base excess [BE]) and lactate (Lac) levels. For each participant, the Simplified Acute Physiology Score (SAPS) II was obtained at admission and the Sequential Organ Failure Assessment (SOFA) score [22] on the study day.

### Free haemoglobin measurement

Arterial blood samples were withdrawn before and 1 hour after transfusion and immediately centrifuged; plasma samples were stored at -70°C for subsequent analysis. In addition, samples were withdrawn from each transfused RBC-unit; the supernatant was obtained by centrifugation and stored at -70°C for subsequent analysis. fHb was quantified in each sample through colorimetric assay using the Drabkin’s reagent (Sigma-Aldrich, Saint Louis, Missouri, USA).

### Microcirculation measurements with sidestream dark-field (SDF) imaging

Sublingual microcirculatory density and flow were monitored using sidestream dark-field (SDF) videomicroscopy (Microscan, Microvision Medical, Amsterdam, the Netherlands) before and 1 hour after transfusion. Details on the SDF imaging technique have been described elsewhere [16, 23]. Videos from 5 different sites (at least 10 sec/site) were recorded at both time points with adequate focus and contrast and every effort was made to avoid movement and pressure artefacts. Poor-quality images were discarded and 3 images for each time point were selected and analysed using a computer software package (Automated Vascular Analysis Software, Microvision Medical BV, Amsterdam, The Netherlands). According to the consensus report on the performance and evaluation of microcirculation using SDF imaging [24], total vessel density (TVD) and perfused vessel density (PVD) were calculated for small vessels (diameter <20 μm). The De Backer score was calculated as described previously [24]. The proportion of perfused vessels (PPV) and the microvascular flow index (MFI), reflecting microcirculatory blood flow velocity, were analysed semi-quantitatively in small vessels, as described elsewhere [25]. The flow heterogeneity index (HI) was also calculated as the highest MFI minus the lowest MFI divided by the mean MFI, providing an index of heterogeneous microcirculatory perfusion.

In addition, SDF videos were automatically analysed using the GlycoCheck ICU software package (Maastricht University Medical Center, Maastricht, The Netherlands) in order to measure vascular lumen perfused boundary region (PBR). The PBR is considered an index of the dimension of the permeable part of the endothelial glycocalyx which allows the penetration of flowing RBCs [16, 26, 27]. Erythrocytes usually have limited access into an intact glycocalyx, when this is compromised and starts losing its protective capacity, its permeability increases, allowing circulating cells to approximate the luminal endothelial membrane. As a result, the dimension of the erythrocyte PBR will increase. A detailed description of this methodology can be found elsewhere [28].

### Peripheral O_2_ and Hb measurements with near-infrared spectroscopy (NIRS)

Before and 1 hour after transfusion, near-infrared reflectance spectrophotometry (InSpectra™ Model 650; Hutchinson Technology Inc., Hutchinson, MN, USA) was used on the thenar emincence to measure peripheral tissue oxygen saturation (StO_2_) and tissue Hb index (THI) [29, 30] at baseline and during a vascular occlusion test (VOT), using a 40% StO2 target for the ischemic phase [16, 31]. StO₂ was continuously recorded during the reperfusion phase until stabilization [32]. The StO₂ downslope (%/minute) was calculated from the regression line of the first minute of StO₂ decay after occlusion, providing an index of O₂ consumption rate. The StO₂ upslope (%/minute) was obtained from the regression line of StO₂ increase in the reperfusion phase. The area under the curve (AUC) of the hyperaemic response was also calculated. StO₂ upslope and the area under the curve (AUC) StO₂ reflect microvascular reactivity [32]. All the parameters were calculated using a computer software package (Version 3.03 InSpectra Analysis Program; Hutchinson Technology Inc.).

### Sample size calculation and statistical analysis

The sample size had been originally calculated on the basis of MFI data (primary endpoint) [16]. The secondary analysis presented herein focused on a different objective (changes in plasma free Hb levels). The power of this analysis was assessed *a posteriori*.

Statistical analysis was performed using GraphPad Prism version 5 (GraphPad Software, La Jolla, CA). A Mann Whitney U test was used to evaluate differences between the two groups at baseline and after blood transfusion. Wilcoxon matched-pairs signed rank test was used for comparative analysis of data sets obtained before and 1 hour after RBC transfusion. A Spearman coefficient was evaluated to study the correlation between variables. In a supplementary analysis, non-normally distributed data were normalized whenever possible through logarithmic or reciprocal transformation and a two-way analysis of variance (ANOVA) for repeated measures was performed with Bonferroni post-hoc test in order to compare changes in the parameters of interest between the two groups. Data are presented as median (25^th^-75^th^ percentiles), unless otherwise indicated. Differences were considered significant at p values <0.05.

## Results

Twenty patients were studied in total (10 patients per group). Patient characteristics are shown in Table 1. Sixteen patients out of 20 received 2 blood units, 3 patients received only 1 RBC-unit (1 patient in the fresh RBC group, 2 in the old RBC group) and 1 patients in the fresh RBC group received 3 blood units. No other blood components were given during the study period. Storage was 4 [3.5–5] days in the fresh RBC group and 30 [22–30] days in the old RBC group. None of the included patients had a medical history of hemoglobinopathies, erythrocyte membrane defects, enzymatic defects of microangiopathies or any other disease that could induce hemolysis thus influencing plasma fHb levels.

**Table 1:** Patient characteristics for the two groups.

	Fresh RBCs^a^	Old RBCs
	(n = 10)	(n = 10)
Age, years	70 (65–72)	70 (47–79)
Sex (male; female)	5; 5	6; 4
SAPS II (on admission)	37 (28–74)	30 (25–40)
ICU days before enrollment	9 (8–12)	7 (5–11)
SOFA score	8 (5–12)	6 (3–8)
Sepsis (n)	2	5
Severe sepsis (n)	3	1
Septic shock (n)	5	4
Source of infection (n)		
lung	4	5
abdomen	1	1
urinary tract	2	0
miscellaneous	3	4
Adrenergic dose^b^		
Norepinephrine^b^	5; 0.047 (0.015–0.370)	4; 0.086 (0.074–0.234)
Dobutamine^b^	1; 2.074	0

RBCs = red blood cells; SAPS II = Simplified Acute Physiology Score II; ICU = Intensive Care Unit; SOFA = Simplified Organ Failure Assessment.

Data are expressed as median (interquartile range) unless stated otherwise. Sepsis, severe sepsis, septic shock are reported as independent categories.

^a^These data have been already presented in [16] as “non-leukodepleted group”.

^b^number of patients; dose in μg/kg*min [median (interquartile range)].

### Hematologic, hemodynamic and gas exchange variables

Hematologic, hemodynamic and gas exchange variables before and 1 hour after transfusion in the two groups are presented in Table 2. Baseline differences were found between the two groups: MAP was higher and lactate levels lower in the old RBC group (p<0.001 and p<0.01, respectively). Hb and Hct were elevated after transfusion in both groups (p<0.01 in all cases). MAP increased after transfusion in the fresh RBCs group (p = 0.04). BE decreased in both groups. Lactate levels differed significantly after transfusion between the two groups (p<0.01). Results of two-way ANOVA are reported in S1 Table.

**Table 2:** Hematologic, hemodynamic and gas exchange variables in the two groups (baseline and 1 hour after transfusion).

	Fresh RBCs^a^			Old RBCs			before-after changes, between-group comparison
	(n = 10)			(n = 10)			
	*before*	*after*	*p* ^*b*^	*before*	*after*	*p* ^*b*^	*p* ^*c*^
Hb (g/dL)	8.4 (7.9–8.8)	10.4 (9.9–11.5)	<0.01	8.6 (8.5–8.8)	10.4 (10.0–10.8)	<0.01	0.47
Hct (%)	26.7 (26.0–28.0)	32.5 (29.9–34.6)	<0.01	27.3 (26.4–28.7)	32.1 (31.0–34.4)	<0.01	0.28
HR (bpm)	72 (59–98)	70 (60–86)	0.20	81 (72–98)	77 (74–94)	0.86	0.34
MAP (mmHg)	70 (67–77)	77 (72–98)	0.04	96 (80–111) ^ddd^	92 (84–119)	0.91	0.23
Urine output (mL/d)	3145 (2516–3625)	3572 (2385–4266)	0.99	3273 (2466–3933)	3212 (2315–3793)	0.06	0.33
T (°C)	36.8 (35.9–37.3)	36.7 (36.1–37.4)	0.48	36.9 (36.5–37.4)	37.0 (36.8–37.6)	0.16	0.73
WBC (x10^3/mcL)	11.9 (5.2–17.3)	12.5 (5.1–17.2)	0.99	14.5 (11.9–18.3)	14.4 (11.3–17.2)	0.69	0.66
PLT (x10^3/mcL)	190 (112–225)	198 (87–216)	0.86	236 (146–268)	270 (167–218)	0.21	0.14
pH	7.48 (7.36–7.54)	7.49 (7.37–7.52)	0.07	7.49 (7.46–7.50)	7.47 (7.45–7.48)	0.07	0.73
PaO2 (mmHg)	123 (103–149)	105 (96–125)	0.06	129 (97–174)	123 (111–135)	0.32	0.88
PaCO2 (mmHg)	42 (36–45)	42 (37–46)	0.94	40 (37–43)	41 (38–43)	0.28	0.88
PaO2/FiO2	230 (206–309)	215 (173–298)	0.36	269 (230–403)	284 (236–353)	0.62	0.73
BE (mEq/L)	5.7 (1.8–10.5)	5.3 (1.3–9.5)	0.03	5.9 (4.3–8.5)	5.6 (2.7–8.0)	0.05	0.76
Lac (mmol/L)	1.2 (0.9–1.7)	1.3 (1.0–1.8)	0.44	0.7 (0.6–1.0) ^dd^	0.7 (0.7–1) ^dd^	0.93	0.88

^a^These data have been already presented in [16] as “non-leukodepleted group”.

^b^pre vs. post, Wilcoxon matched-pairs signed rank test.

^c^ between-group comparison of delta [after-before] values, Mann-Whitney U test.

^dd^p<0.01

^ddd^p<0.001, vs. fresh RBCs group at the same time point, Mann-Whitney U test.

Data are expressed as median (interquartile range).

RBCs = red blood cells; Hb = haemoglobin; Hct = haematocrit; HR = heart rate; MAP = mean arterial pressure; T = body temperature; WBC = white blood cell count; PLT = platelet count; BE = base excess; Lac = arterial lactate levels.

### Free hemoglobin

fHb levels in the supernatant of blood units did not differ between fresh and old RBCs (0.103 [0.073–0.149] mg/mL in fresh RBC-units, 0.111 [0.070–0.187] mg/mL in old RBC-units, p = 0.4). No correlation was found between the age of the transfused RBC-units and fHb levels in the supernatant (r = 0.03 [95% CI -0.46, 0.49], p = 0.9; data not shown).

**Fig 1:**
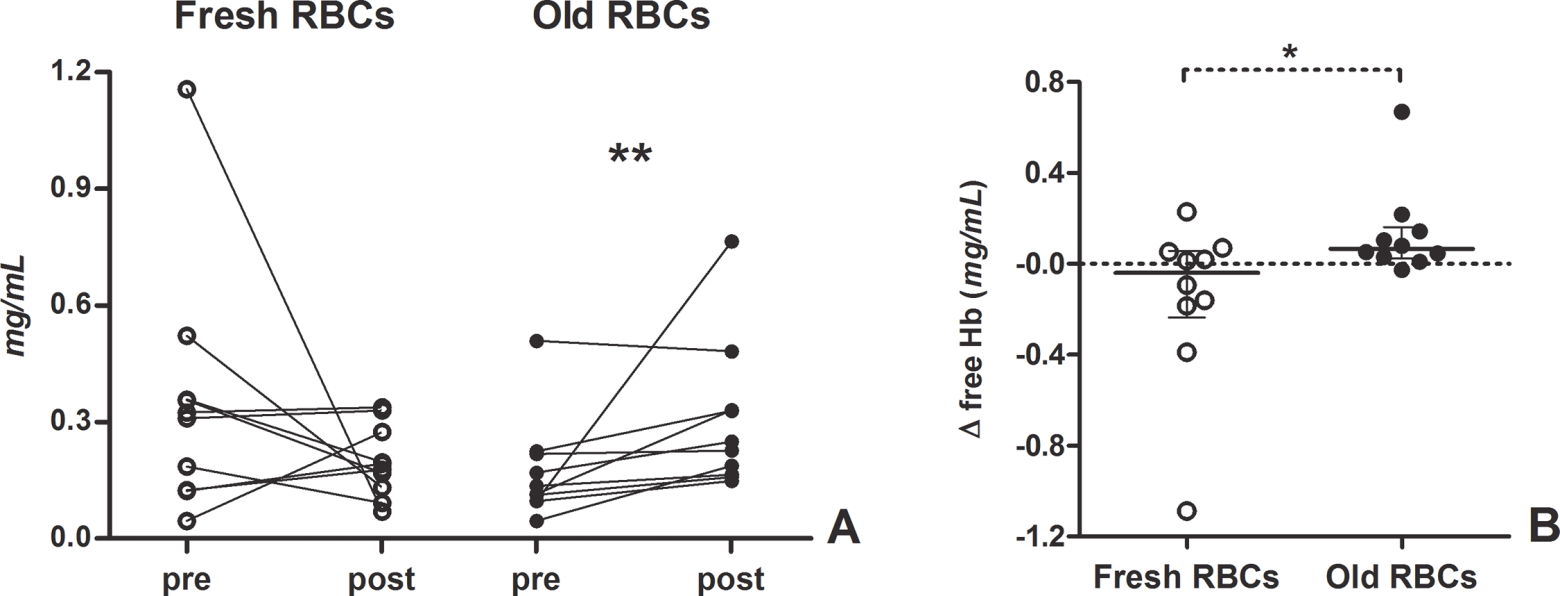
Changes in plasma free haemoglobin after blood transfusion in the two groups. (A) Individual changes in plasma free haemoglobin after blood transfusion in the two groups; **p<0.01, Wilcoxon matched-pair signed rank test. (B) Delta values (after-before transfusion) of plasma free haemoglobin in the two groups. *p<0.05, Mann-Whitney U test. Open circles indicate patients in the fresh RBC group, full circles patients in the old RBC group.

Baseline plasma fHb levels did not significantly differ between the two groups (p = 0.07). Plasma fHb was elevated after transfusion only in old RBCs group (Table 3, Fig 1A). No difference was found in plasma fHb after transfusion between the two groups (p = 0.28). Changes in plasma fHb (delta fHb [before-after transfusion]) differed between the two groups (-0.041 [-0.237, -0.057] mg/mL in the fresh RBCs group, 0.065 [0.024–0.161] mg/mL in the old RBCs group, p = 0.04, Fig 1B). Two-way ANOVA showed a significant interaction between time and type of RBCs transfused, without revealing however any significant difference between the two groups at each time point (S1 Table and S1 Fig).

**Table 3:** Plasma free haemoglobin, sublingual microvascular parameters and NIRS-derived variables in the two groups (baseline and 1 hour after blood transfusion).

	Fresh RBCs^a^			Old RBCs			before-after changes, between-group comparison
	(n = 10)			(n = 10)			
	*before*	*after*	*p* ^*b*^	*before*	*after*	*p* ^*b*^	*p* ^*c*^
Free Haemoglobin (mg/mL)	0.317 (0.124–0.398)	0.185 (0.122–0.288)	0.37	0.125 (0.098–0.219)	0.238 (0.163–0.369)	<0.01	0.04
MFI [AU]	2.75 (2.43–2.87)	2.62 (2.38–3.00)	0.73	2.79 (2.50–3.00)	2.98 (2.69–3.00)	0.55	0.21
De Backer score (1/mm)	11.3 (9.7–11.8)	11.3 (9.3–14.4)	0.16	11.2 (10.5–12.1)	11.8 (10.1–12.1)	0.85	0.39
TVD (mm/mm²)	18.4 (16.6–19.8)	19.6 (15.1–23.3)	0.19	16.8 (13.1–19.7)	17.2 (13.5–19.0)	0.99	0.53
PVD (mm/mm²)	16.2 (14.3–17.7)	17.6 (13.5–21.4)	0.23	15.1 (11.8–17.6)	16.5 (12.0–17.7)	0.23	0.99
PPV (%)	88.5 (83.1–93.1)	90.6 (85.8–96.6)	0.32	90.3 (86.3–96.3)	95.7 (93.3–97.1)	0.11	0.48
HI	0.23 (0.13–0.50)	0.16 (0.00–0.32)	0.31	0.18 (0.00–0.28)	0.02 (0.00–0.28)	0.47	0.82
THI [AU]	10.5 (7.8–11.2)	13.4 (10.4–15.8)	<0.01	11.4 (9.7–12.6)	13.0 (9.1–15.9)	0.05	0.08
StO₂ (%)	88 (80–90)	90 (85–93)	0.03	82 (76–87)	84 (75–93)	0.40	0.67
StO₂ down (%/min)	(-)9.5 [(-)11(-)8.5]	(-)9.0 [(-)10.4(-)7.5]	0.03	(-)9.6 [(-)18.4 (-)7.3]	(-)8.8 [(-)14.5 (-)7.0]	0.16	0.97
StO₂ up (%/min)	174 (81–220)	191 (133–242)	0.01	159 (96–192)	182 (139–220)	0.32	0.68
AUC StO₂ (%*min)	10.7 (8.4–21)	10.9 (8.2–25.4)	0.99	15.1 (4.6–24.0)	14.3 (5.8–24.1)	0.92	0.97
PBR (μm)	2.69 (2.53–2.94)	2.72 (2.65–2.86)	0.23	2.68 (2.65–2.91)	2.77 (2.58–2.93)	0.84	0.62

^a^These data have been already presented in part in [16] as “non-leukodepleted group”.

^b^pre vs. post, Wilcoxon matched-pairs signed rank test.

^c^between-group comparison of delta [after-before] values, Mann-Whitney U test.

Data are expressed as median (interquartile range).

RBCs = red blood cells; MFI = microvascular flow index; TVD = total vessel density; PVD = perfused vessel density; PPV = proportion of perfused vessels; HI = flow heterogeneity index; THI = tissue hemoglobin index; StO2 = tissue oxygen saturation; AUC StO2 = area under the curve of the StO2 (reactive hyperemia following the vascular occlusion test); PBR = perfused boundary region. MFI, TVD, PVD, PPV and HI were calculated in small vessels (diameter <20 μm).

A *post-hoc* analysis showed that our study with a sample of 10 patients per group was able to demonstrate the observed change in fHb after blood transfusion with a power >90% (type II error of 0.06).

### Microvascular response to fresh or old RBC transfusion

Sublingual microvascular parameters and NIRS-derived variables before and 1 hour after transfusion in the two groups are presented in Table 3.

No difference was found in baseline values between the groups. We could not find any significant change in MFI, PPV, TVD, PVD, De Backer score, HI and PBR after the transfusion of either fresh or old RBCs. The change in PPV after transfusion was inversely related to the baseline PPV value in the whole sample (r = -0.53 [95% CI -0.79–0.10], p = 0.02; data not shown). Changes in TVD, PVD and De Backer score were not correlated to their baseline values (r = 0.18 [95% CI -0.30, 0.59] p = 0.4, r = 0.03 [95% CI -0.43, 0.48] p = 0.9, r = -0.21 [95% CI -0.61, 0.26] p = 0.4, respectively).

StO_2_, StO_2_ downslope and StO_2_ upslope increased in the fresh RBCs group. THI was elevated after transfusion in both groups. The AUC StO_2_ remained unaltered in both groups. Changes in NIRS-derived variables were not correlated to their baseline values.

All SDF- and NIRS-derived parameters did not differ after transfusion between the groups. Two-way ANOVA did not show any significant effect of the type of transfused RBCs on the changes in SDF- or NIRS-derived parameters after blood transfusion (S1 Table).

No correlation was found between baseline SOFA score and microvascular changes after blood transfusions. The change in MFIs tended to correlate with the baseline MAP (r = 0.47 [95% CI, 0.01–0.77], p = 0.04).

### Free haemoglobin and microcirculation

**Fig 2:**
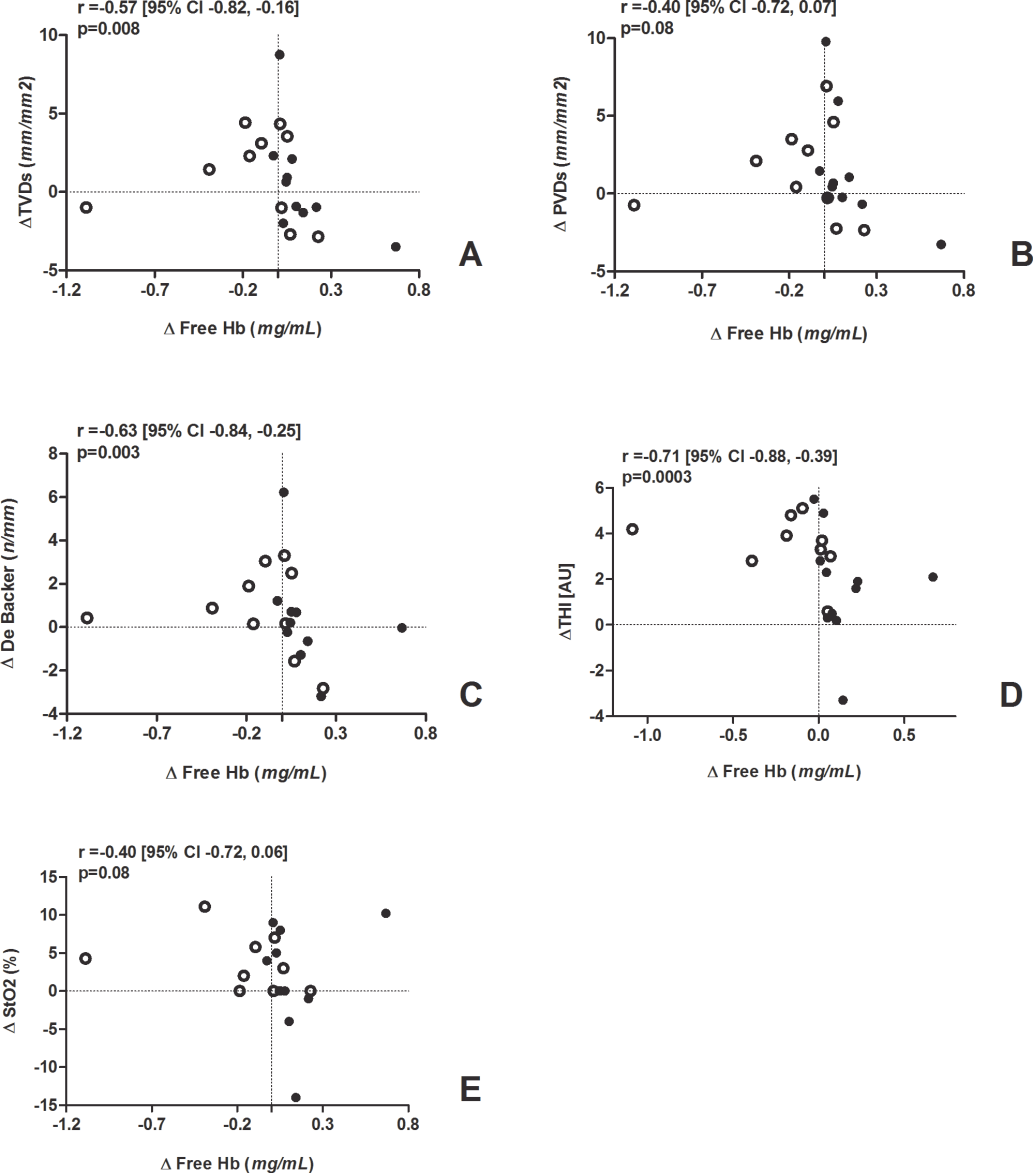
Correlation analysis between the change in plasma fHb (X axis) and changes in: (A) total small vessel density, (B) perfused vessel density, (C) De Backer score, (D) tissue haemoglobin index, (E) tissue oxygen saturation (Y axis).

The change in fHb (delta fHb [after-before transfusion]) was negatively correlated with changes in TVD (r = -0.57 [95% CI -0.82, -0.16], p = 0.008), De Backer score (r = -0.63 [95% CI -0.84, -0.25], p = 0.003) and THI (r = -0.71 [95% CI -0.88, -0.39], p = 0.0003) (Fig 2 and S2 Fig). The change in PVD and StO_2_ tended to be inversely correlated with the change in fHb (r = -0.40 [95% CI -0.72, 0.07], p = 0.08 and r = -0.40 [95% CI -0.72, 0.06], p = 0.08, respectively) (Fig 2B–2E and S2 Fig). Changes in MFI, PPV, StO2 upslope, StO2 downslope and AUC StO2 were not correlated with the change in fHb.

Open circles indicate patients in the fresh RBC group, full circles patients in the old RBC group.

## Discussion

In the present study, the transfusion of old RBCs was associated with an increase in plasma fHb in septic patients. We were not able to demonstrate any improvement in the microcirculation after transfusion in either group, nor did we find any clear difference in the microcirculatory response to the transfusion of fresh versus old RBCs. The change in plasma fHb after transfusion was negatively correlated with changes in sublingual microvascular density and peripheral tissue Hb content.

Increasing fHb concentration in the supernatant of packed RBCs has been described as a function of time during blood storage [8, 33], and the transfusion of 2 packed RBC units increased circulating fHb levels in patients with hematologic malignancies [33]. In the present study, septic patients who received old blood transfusions showed an increase in plasma fHb, despite the absence of significantly higher fHb levels in the supernatant of old blood units. It is possible that the transfusion of older fragile erythrocytes led to premature intravascular RBC rupture in the recipients. We cannot exclude that the underlying critical illness had influenced our results: spontaneous changes unrelated to blood transfusion might reasonably explain the decrease in fHb observed in 6 patients out of 20.

RBC storage lesions can be responsible for the association between the transfusion of older blood and adverse outcomes [34]. Old RBCs decreased microvascular oxygenation and flow in rat isovolemic exchange models [35, 36]. Nonetheless, clinical data remain controversial [20, 37–39]. Marik et al. were the first to report a harmful effect of duration of RBC storage on systemic and tissue oxygenation in septic patients [40], but this association was not confirmed [41]. RBC storage time showed no influence on the sublingual microvascular response to the transfusion of leukodepleted blood in patients with severe sepsis [19]. In the present study, the transfusion of non-leukodepleted blood did not apparently affect the sublingual microcirculation and no difference could be seen in the microvascular response to the transfusion of fresh versus old RBCs. Whereas both fresh and old RBC transfusions were able to increase the peripheral tissue Hb content, StO_2_, StO_2_ downslope and StO_2_ upslope increased significantly only in the fresh RBC group. Of note however, the old RBC group showed similar absolute changes: the study may have been underpowered to detect significant differences in these parameters.

We found an inverse relationship between the changes in the microcirculation and the change in plasma fHb. Independently of the age of the transfused blood or the baseline microvascular status, an increase in circulating fHb after transfusion was associated with a decrease in the sublingual microvascular density and a lower increase in peripheral tissue Hb content. We can speculate that these effects were mediated by disturbances in NO metabolism: cell-fHb can react with NO much faster than RBC-encapsulated Hb [42] and inhibit the NO-mediated vasodilation. Experimental studies showed that the infusion of the supernatant from stored RBCs produces potent vasoconstriction that is correlated with the amount of fHb in the storage medium [8]. Blood transfusion increased NO consumption in patients with hematologic malignancies [33]. Our results are consistent with these findings. Sepsis-induced deregulation in NO production is associated with impaired microvascular perfusion and reduced O_2_ consumption [11]. Although inhibiting NO during sepsis increases blood pressure, it also reduces microvascular blood flow and exacerbates abnormal oxygen transport [43]. We found an increase in MAP in the fresh RBC group but not in the old RBC group: this would contradict the previous findings. However, the baseline between-group discrepancy may have confounded the results and prevents to draw any conclusion on this point. Increasing NO-scavenging by fHb due to old RBC transfusion may synergize with the underlying endothelial dysfunction, thus reducing NO bioavailability and producing relative vasoconstriction [44]. Notably, we studied stable patients with low baseline microvascular alterations, as indicated by PPV above 70% in all patients and median MFI above 2.6 in both groups [45]: the observed interaction might be more pronounced and deleterious in presence of severe underlying microcirculatory dysfunction. Although our results would suggest that there may be a relationship between the changes in plasma fHb and the microvascular response after transfusion, several points remain to be clarified, namely the role of the patient’s underlying clinical condition and microcirculatory perfusion, and the potential advantages of pre-storage leukodepletion.

The microcirculatory response to blood transfusion is likely to be influenced by the underlying clinical and microvascular condition of the recipient. In our study, the heterogeneity of the studied population, which included patients with different severity of sepsis, may have been a source of variability in the response observed. More importantly, baseline disparities between the two groups (lower MAP and higher lactate levels in the fresh RBC-group) reasonably influenced the results and add to the uncertainty of our data. In fact, a relationship seemed to exist between baseline MAP and the increase in MFI. However, patients who received old RBC transfusions did not show any significant improvement in microvascular convective flow despite higher baseline MAP. Other factors could have played a role. In previous studies, the microvascular response to blood transfusion was negatively correlated with the baseline microcirculatory status rather than the age of the transfused blood units [19, 20]. Accordingly, in the present study the change in PPV was inversely related to its baseline value.

Our analysis was focused on a single aspect of packed-RBC storage lesions; other potentially important factors, such as loss of RBC deformability and accumulation of residual leukocyte-derived cytokines within the storage medium, were not considered. Some studies suggest that pre-storage leukodepletion may abrogate the detrimental effects of packed RBC aging [46]. The real role of inflammatory mediators from residual leukocytes in the development of storage lesions remains to be clarified. Future studies should investigate whether pre-storage leukodepletion may really preserve the integrity of stored RBCs and prevent the release of fHb.

The first limitation of the present study is that it is a secondary analysis of a randomized pilot study with a different primary endpoint. Moreover, we enrolled a small number of patients. However, a *post-hoc* analysis showed that our study had a power >90% to detect the observed variation in plasma fHb, which was the main objective of this investigation. The heterogeneity of the studied patient population may have been a source of variability, thus preventing to detect different effects of fresh versus old RBC transfusions. In addition, baseline differences between the two groups reasonably influenced the microvascular response observed and impeded a proper between-group comparison. Another limitation of our analysis is the fact that a large number of statistical tests was performed on a small sample size; it is possible that some of the observed associations were due to chance. Our investigation was designed as a pilot study aimed to detect any possible relationship between the type of transfused blood and changes in plasma fHb and/or microcirculation: therefore, a higher type I error rate was deemed acceptable and a correction for multiple comparisons was not applied as it would have substantially reduced the probability of finding any statistically significant associations [47]. A two-way ANOVA with Bonferroni post-hoc test performed after normalization of the data revealed a significant interaction between the type of transfused RBCs and the changes in plasma fHb after transfusion. However, our results are not conclusive and require validation from larger studies.

In our previous report [16], we explored the impact of blood transfusion on the endothelial glycocalyx. Unfortunately, it was not possible to evaluate the relationship between variations in plasma fHb and markers of glycocalyx disruption (hyaluronan, syndecan-1, heparan sulphate) as these were not measured for the old RBC group due to cost reasons. Since the glycocalyx plays a major role in the shear stress-induced release of NO [48, 49], future studies should be addressed to investigate its impact on NO bioavailability after the transfusion of stored blood.

Finally, only non-leukodepleted RBCs were used: this may limit the direct applicability of our results, as most developed countries currently use leukodepleted blood units. Nevertheless, the use of non-leukodepleted RBCs is still the standard practice in Italy, and universal leukoreduction has not been implemented yet in several countries including USA.

## Conclusions

In the present study, the transfusion of old RBCs was associated with an increase in plasma fHb in a small and heterogeneous population of septic patients. The sublingual microcirculation appeared globally unaffected by the transfusion of either fresh or old RBCs. Independently of the type of blood received and the baseline microvascular status, increasing plasma fHb levels after transfusion were associated with decreasing sublingual microcirculatory density and lower increase in peripheral tissue Hb content after transfusion. Further studies are needed to confirm these findings.

## Supporting Information

S1 Fig Two-way analysis of variance for repeated measures of the primary outcome of interest freeHb. Data were normalized through base-10 logarithm transformation and are expressed as mean and 95% confidence interval. Two-way ANOVA for repeated measures showed a significant interaction between time point and type of transfused RBCs. Bonferroni post-hoc tests revealed no significant differences between the groups at each time point.
(TIF)

S2 Fig Negative correlations between the change in plasma fHb (X axis) and changes in: (A) total small vessel density, (B) perfused vessel density, (C) De Backer score, (D) tissue haemoglobin index, (F) tissue oxygen saturation (Y axis). Data are expressed as ranks. Open circles indicate patients in the fresh RBC group, full circles patients in the old RBC group.
(JPG)

S1 Table Two-way analysis of variance for repeated measures. Normality of distribution was checked through the Shapiro-Wilk normality test. Non-normally distributed variables (fHb, MFIs, De Backer score, PVD, StO_2_ downslope, AUC StO_2_, HR, PaO_2_/FiO_2_) were normalized whenever possible through logarithmic or reciprocal transformation as appropriate. Data were analyzed through a two-way analysis of variance for repeated measures with Bonferroni post-hoc test. A p value <0.05 was used to indicate statistical significance. The variables Microcirculatory Flow Index and Flow Heterogeneity Index could not be normalized and the parametric statistics was not applied in these cases.
(PDF)
