# Supplementary figures and images for: Plasma Free Hemoglobin and Microcirculatory Response to Fresh or Old Blood Transfusions in Sepsis
Source: PLoS One. 2015 May 1;10(5):e0122655. doi: 10.1371/journal.pone.0122655 (PMC4416810; doi:10.1371/journal.pone.0122655)

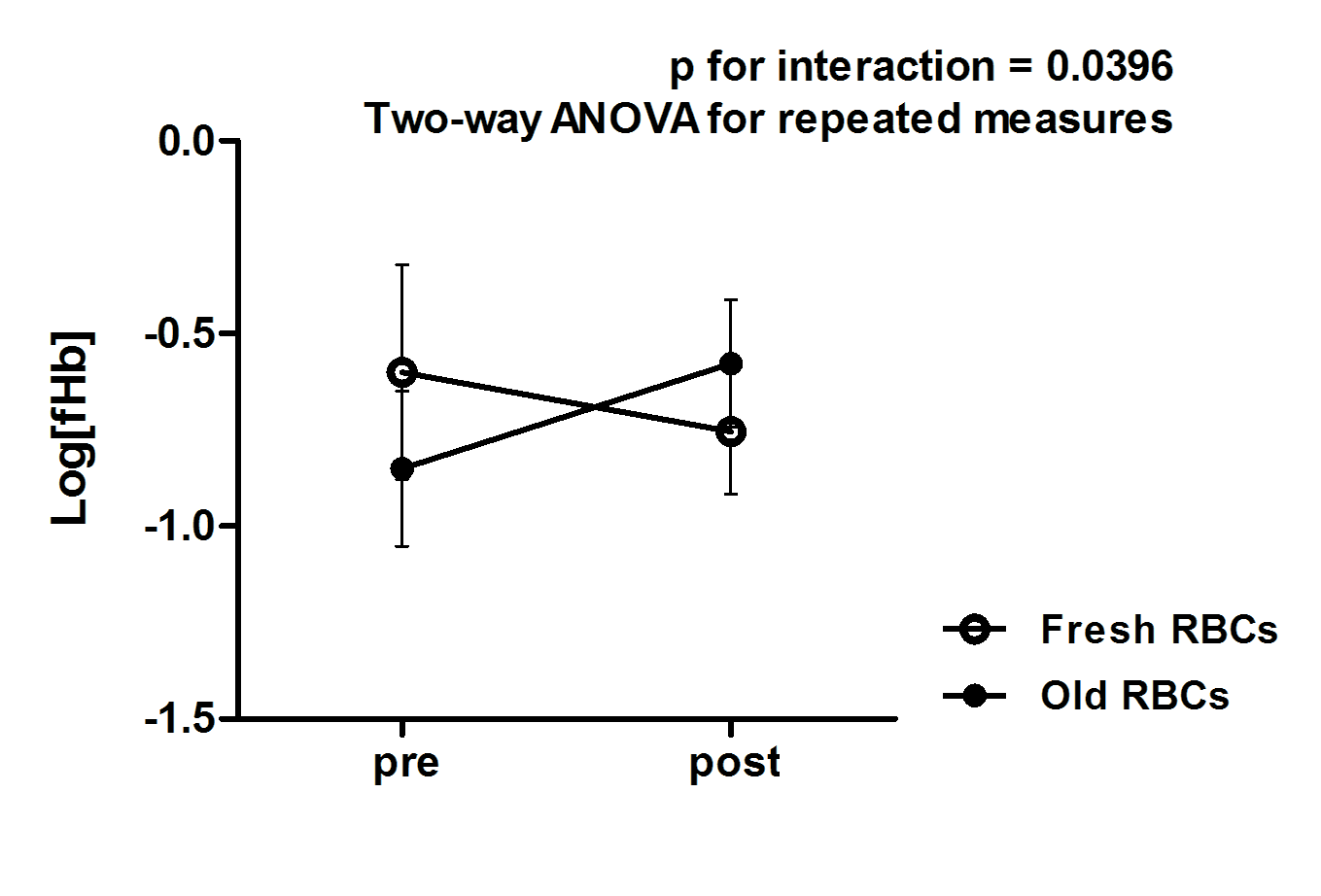

Supplement: S1 Fig — Data were normalized through base-10 logarithm transformation and are expressed as mean and 95% confidence interval. Two-way ANOVA for repeated measures showed a significant interaction between time point and type of transfused RBCs. Bonferroni post-hoc tests revealed no significant differences between the groups at each time point. (TIF) [file pone.0122655.s001.tif]

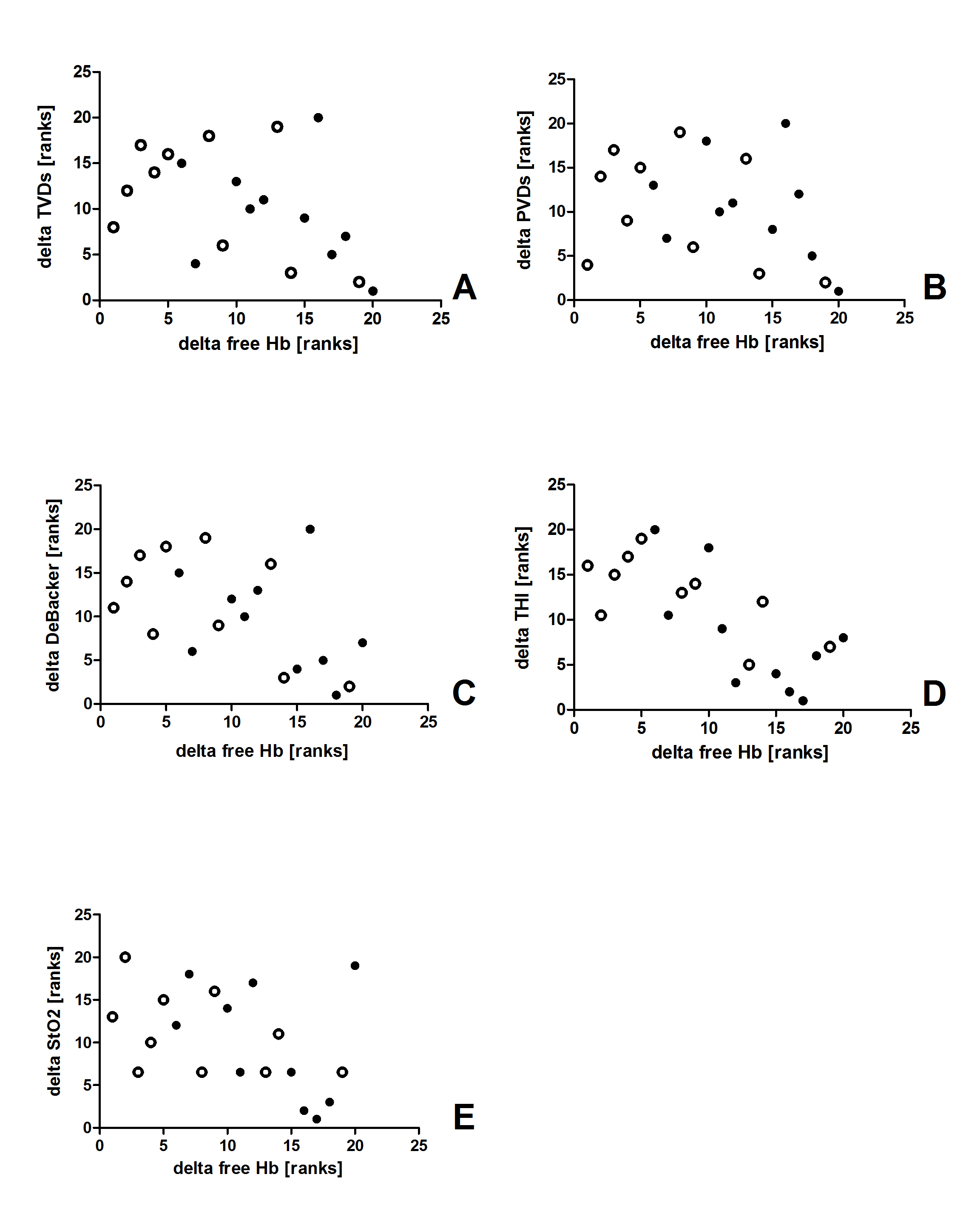

Supplement: S2 Fig — Data are expressed as ranks. Open circles indicate patients in the fresh RBC group, full circles patients in the old RBC group. (JPG) [file pone.0122655.s002.jpg]
