# Supplementary material for: Plasma Free Hemoglobin and Microcirculatory Response to Fresh or Old Blood Transfusions in Sepsis
Source: PLoS One. 2015 May 1;10(5):e0122655. doi: 10.1371/journal.pone.0122655 (PMC4416810; doi:10.1371/journal.pone.0122655)
Supplement: S1 Table — Normality of distribution was checked through the Shapiro-Wilk normality test. Non-normally distributed variables (fHb, MFIs, De Backer score, PVD, StO2 downslope, AUC StO2, HR, PaO2/FiO2) were normalized whenever possible through logarithmic or reciprocal transformation as appropriate. Data were analyzed through a two-way analysis of variance for repeated measures with Bonferroni post-hoc test. A p value <0.05 was used to indicate statistical significance. The variables Microcirculatory Flow Index and Flow Heterogeneity Index could not be normalized and the parametric statistics was not applied in these cases. (PDF) [file pone.0122655.s003.pdf]

Table S1 – Two-way analysis of variance for repeated measures.

Normality of distribution was checked through the Shapiro-Wilk normality test. Non-normally distributed variables (fHb, MFIs, De Backer score, PVD, StO_2 downslope, AUC StO_2, HR, PaO_2/FiO_2) were normalized whenever possible through logarithmic or reciprocal transformation as appropriate. Data were analyzed through a two-way analysis of variance for repeated measures with Bonferroni post-hoc test. A p value <0.05 was used to indicate statistical significance. The variables Microcirculatory Flow Index and Flow Heterogeneity Index could not be normalized and the parametric statistics was not applied in these cases.

	p for interaction	*p for time point*	*p for type of RBCs*	*Bonferroni post-hoc test*
Hemoglobin	0.366	<0.001	0.966	n.s.
Hematocrit	0.436	<0.001	0.719	n.s.
Heart rate	0.413	0.162	0.255	n.s.
Mean Arterial Pressure	0.197	0.135	0.005	p<0.01, fresh Vs. old RBCs before transfusion
Urine Output	0.317	0.612	0.979	n.s.
Temperature	0.578	0.130	0.496	n.s.
White Blood Cells	0.547	0.898	0.341	n.s.
Platelets	0.114	0.171	0.601	n.s.
pH	0.590	0.007	0.503	n.s.
PaO2	0.614	0.053	0.178	n.s.
PaCO2	0.731	0.590	0.625	n.s.
PaO2/FiO2	0.878	0.403	0.087	n.s.
Base Excess	0.505	0.002	0.648	n.s.
Lactate	0.802	0.241	<0.001	p<0.01, fresh Vs. old RBCs before or after transfusion
Free Hemoglobin	0.0396	0.545	0.670	n.s.
Microcirculatory Flow Index (small vessels)	n.a.	n.a.	n.a.	n.a.
De Backer score	0.623	0.491	0.714	n.s.
Total small Vessel Density	0.695	0.223	0.247	n.s.
Perfused small Vessel Density	0.863	0.080	0.356	n.s.
Proportion of Perfused small Vessels	0.324	0.053	0.248	n.s.
Flow Heterogeneity Index	n.a.	n.a.	n.a.	n.a.
Perfused Boundary Region	0.652	0.352	0.693	n.s.
StO2	0.543	0.067	0.161	n.s.
StO2 downslope	0.521	0.019	0.517	n.s.
StO2 upslope	0.838	0.021	0.705	n.s.
Area under the curve StO2	0.971	0.574	0.765	n.s.
Tissue Hemoglobin Index	0.086	<0.001	0.618	n.s.

n.s. not significant, n.a. not applicable
